# Supplementary material for: Investigating the Effects of Seizures on Procedural Memory Performance in Patients with Epilepsy
Source: Brain Sci. 2021 Feb 19;11(2):261. doi: 10.3390/brainsci11020261 (PMC7922212; doi:10.3390/brainsci11020261)
Supplement: Supplementary file 1 [file brainsci-11-00261-s001.pdf]

Supplementary material

Investigating the Effects of Seizures on Procedural Memory Performance in Patients with Epilepsy

Frank J. van Schalkwijk[1], Walter R. Gruber[2], Laurie A. Miller[3,4], Eugen Trinka[1], and Yvonne Höller[1,5,*].

[1] Department of Neurology, Christian Doppler Medical Centre and Centre for Cognitive Neuroscience, Paracelsus Medical
University, 5020 Salzburg, Austria; frankjasper.vanschalkwijk@sbg.ac.at (F.J.v.S); e.trinka@salk.at (E.T.)

[2] Department of Psychology, Centre for Cognitive Neuroscience (CCNS), University of Salzburg, 5020 Salzburg, Austria; walter.r.gruber@sbg.ac.at (W.R.G.)

[3] Institute of Clinical Neurosciences, Royal Prince Alfred Hospital and Central Medical School, University of Sydney, Camperdown NSW 2050, Sydney, Australia; laurie.miller@sydney.edu.au (L.A.M.)

[4] ARC Centre of Excellence in Cognition and Its Disorders, University of Sydney, Camperdown NSW 2006, Sydney,
Australia.

[5] Faculty of Psychology, University of Akureyri, 600 Akureyri, Iceland; yvonne@unak.is (Y.H.)

[*] Correspondence: yvonne@unak.is; Tel.: +35 (0) 446 085 76

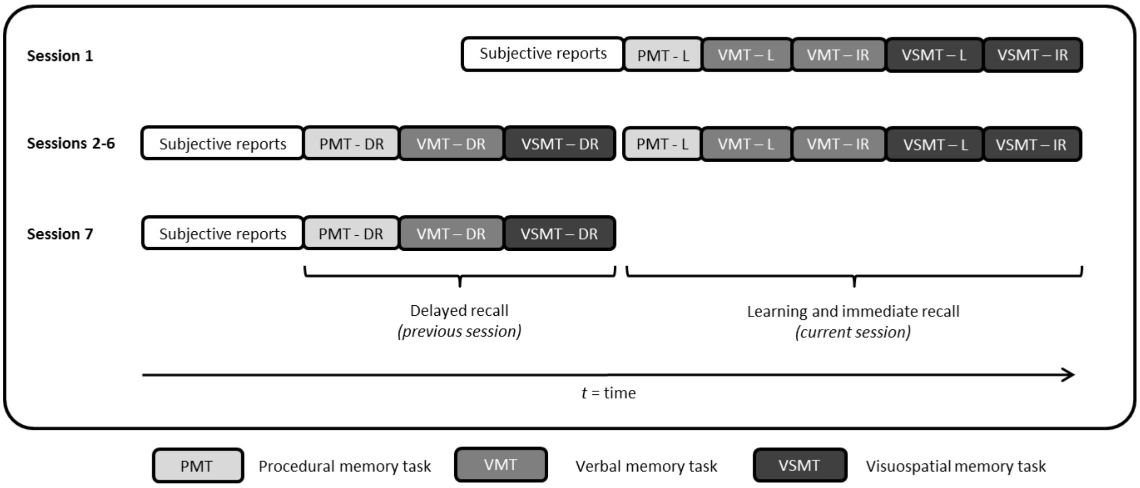

Figure S1. Study protocol – extended. This figure is a more detailed illustration of the timeline per session, supplementing Figure 1 in the main manuscript. Each session started with subjective reports of (potential) sleep duration prior to the start of the session, as well as current experiences of fatigue, stress, and the ability to concentrate. The order of memory tasks was kept constant for all patients. The first session only included learning (L) sessions for the procedural memory task (PMT; fingertapping task [12 trials]), verbal memory task (VMT; word-pair association [60 word pairs]), and visuospatial memory task (VSMT [1 virtual town]). Learning sessions for VMT and VSMT were followed by immediate recall (IR). Sessions 2-6 started with delayed recall (DR) for PMT, SMT, and EMT from the previous session, after which patients were trained and evaluated on new versions of each task. The final session only included DR for all three memory tasks. Figure included with permission from Höller et al. (2020) [1].

Instruments

Screening. Patients were screened for depression by filling in an electronic adaptation of the German translation of the Beck Depression Inventory (BDI-II; [2]). Sum scores were determined and classified according to recommendations (score < 9 = no depression; score 9-13 = minimal depression; score 14-19 = mild depression; score 20-28 = moderate depression; score > 28 = heavy depression). In addition, patients' chronotype was determined using an electronic adaptation of the German translation of the

Morningness-Eveningness Questionnaire (D-MEQ)[3]. Scores were determined and classified according to recommendations (definite evening type [score < 31]; moderate evening type [score 31-41]; neutral type [score 42–58]; moderate morning type [score 59-69]; definite morning type [score > 69]). Screening indicated that patients were on average minimally depressed (9.64 ± 8.58; range = 0–39; no depression [*n*=28]; light depression [*n*=11]; minimal depression [*n*=11]; heavy depression [*n*=3]) and were predominantly classified as neutral chronotypes (51.13 ± 9.98; range = 27–71; definite evening type [*n*=2]; moderate evening type [*n*=5]; neutral type [*n*=33]; moderate morning type [*n*=10]; definite morning type [*n*=3]). Patients were not excluded based on their reports of depression or chronotype.

Subjective reports. Prior to each session, patients were asked to estimate how long they had slept in the 12h prior to the session, and to evaluate their current state of mind on the topics of fatigue (*"How tired do you feel at the moment?"*; ranging from 0 [*"absolutely not tired"*] to 9 [*"very tired"*), stress (*"How stressed do you feel at the moment?"*; ranging from 0 [*"very relaxed"]* to 9 [*"very stressed"*]), and concentration (*"How difficult is it for you to concentrate right now?"*; ranging from 0 [*"Very easy"*] to 9 [*"Very difficult"*]).

Confounding factors

Age. Given the procedural memory task includes fine motor skills, there was the possibility that younger patients were more adapt at typing compared to older patients. We therefore selected behavioral performance for those sessions during which no seizure was observed (n = 203) for 51 patients. Using the median age as a cutoff (median age = 27 years), patients were subsequently split into "younger" (age < median; n = 23) and "older" patients (age ≥ median; n = 28). Behavioral performance was contrasted between age groups. Younger patients showed higher performance for speed during the learning (Z = -5.12, p < 0.001, d = 0.749, 95% CI [0.463 – 1.035]) and recall (Z = -4.97, p < 0.001, d = 0.749, 95% CI [0.463 – 1.036]) conditions (Figure S3A); a contrast that was also observed for triplets during learning (Z = -4.863, p < 0.001, d = 0.715, 95% CI [0.429 – 1.00])
and recall (Z = -5.06, p < 0.001, d = 0.755, 95% CI [0.468 – 1.04]; Figure S3B). Accuracy was found to be similar during learning (Z = -1.17, p = 0.120), but was higher during recall for younger patients (86.75 ± 13.08) compared with older patients (83.44 ± 12.73, Z = -2.478, p = 0.007, d = 0.256, 95% CI [-0.022 – 0.534]; Figure S3C). Performance change (recall – learning) for speed showed a nonsignificant trend towards a higher improvement for younger patients (1.17 ± 2.91) compared with older patients (0.55 ± 2.78; Z = -1.38, p = 0.084; Figure S3D), which was significantly better for younger patients when evaluated for triplets (Z = -1.721, p = 0.043, d = 0.285, 95% CI [0.007 – 0.563]; Figure S3E). Performance change for accuracy was similar between younger (-0.55 ± 12.18) and older patients (-1.02 ± 13.11; Z = 1.05, p = 0.853; Figure S3F).

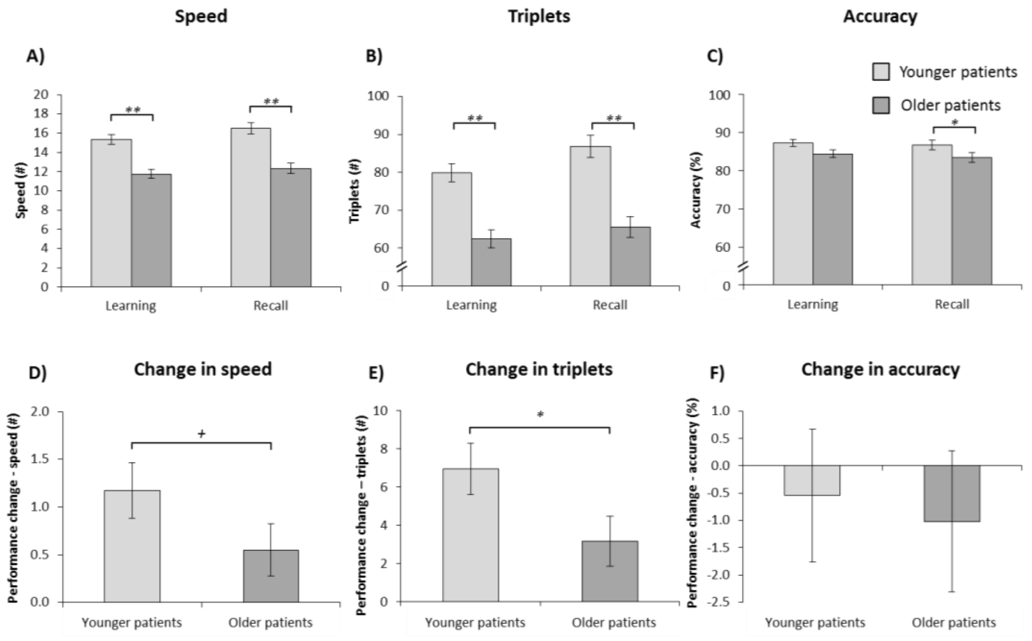

Figure S2. Evaluating the effect of age as a potential confounding factor on behavioral performance (M ± SE). Participants were contrasted based on age group following a median split, revealing better performance for younger patients on **(A)** speed, **(B)** triplets, and **(C)** accuracy during the recall condition. Performance change (recall-learning) trended towards a stronger improvement for younger patients when evaluated for **(D)** speed, whereas performance change for **(E)** triplets was significantly better for younger patients. **(F)** No differences were observed for performance change for accuracy between the two age groups. + $p < 0.100$; * $p < 0.050$; ** $p < 0.010$,

Task difficulty and learning effect. As our paradigm included six retention sessions, the equivalent number of task versions was required. Therefore we used the original fingertapping sequence used by Walker, *et al.* [4] and expanded the task with five other sequences that consisted of five elements (i.e., 1-4-2-3-1, 4-1-3-2-4, 3-2-1-4-3, 2-1-3-4-2, 1-2-4-3-1, and 4-3-1-2-4). As the order of sequences was kept constant between patients, this evaluation of task difficulty also includes the practice effect on the task, meaning that patients were expected to become better due to repeated task execution. It was therefore expected that task performance during the learning condition was lowest on the first version and highest on the last version. As previously stated, performance was evaluated for mean speed, triplets, and accuracy over the last three trials (learning: T10-T12; recall: T2-T4). The potential confound of version difficulty was investigated using data from a subsample of patients who were not excluded based on behavioral outlier criteria, and specifically used behavioral data from patients who participated in all versions ($n = 20$) and who encountered no seizures in any of the sessions.

A repeated-measures ANOVA indicated a main effect of task version during the learning condition for speed ($F_{5, 95} = 3.90$, $p = 0.003$, $\eta_p^2 = 0.17$; Figure S4A) and triplets ($F_{2.99, 56.74} = 5.77$, $p = 0.002$, $\eta_p^2 = 0.23$; Figure S4B) but not accuracy ($F_{3.05, 57.99} = 0.47$, $p = 0.705$, $\eta_p^2 = 0.02$; Figure S4C). Post-hoc Wilcoxon contrasts revealed that speed was higher for version 6 (15.00 ± 4.72) compared to version 1 (13.08 ± 4.45; Z = -2.617, $p = 0.009$); confirming the expected practice effect. In addition, speed was significantly higher for the third version compared to versions 1, 2, 4, and 5 (all $p < 0.038$), whereas no difference was observed compared to version 6 ($Z = -1.027$, $p = 0.304$). For triplets, post-hoc Wilcoxon contrasts revealed that triplets were higher for version 6 (79.35 ± 23.67) compared to version 1 (66.90 ± 21.91; $Z = -3.119$, $p = 0.002$); confirming the practice effect. Furthermore, triplets were significantly higher for the

third version compared to versions 1-5 (all $p < 0.033$), whereas no difference in performance was observed compared to version 6 ($Z = -1.083$, $p = 0.279$).

As this study aimed to investigate the effects of epileptic seizures on memory performance changes, we further investigated a potential main effect of task version on performance change scores (recall–learning). No significant main effects of task version was observed for the change in speed ($F_{5, 95} = 1.501$, $p = 0.197$; Figure S4D), triplets ($F_{5, 95} = 1.501$, $p = 0.197$; Figure S4E), or accuracy ($F_{2.98, 56.60} = 2.359$, $p = 0.082$; Figure S4F). As performance changes from learning to recall were similar for the six different task versions, task difficulty was not included as a confound in subsequent analyses.

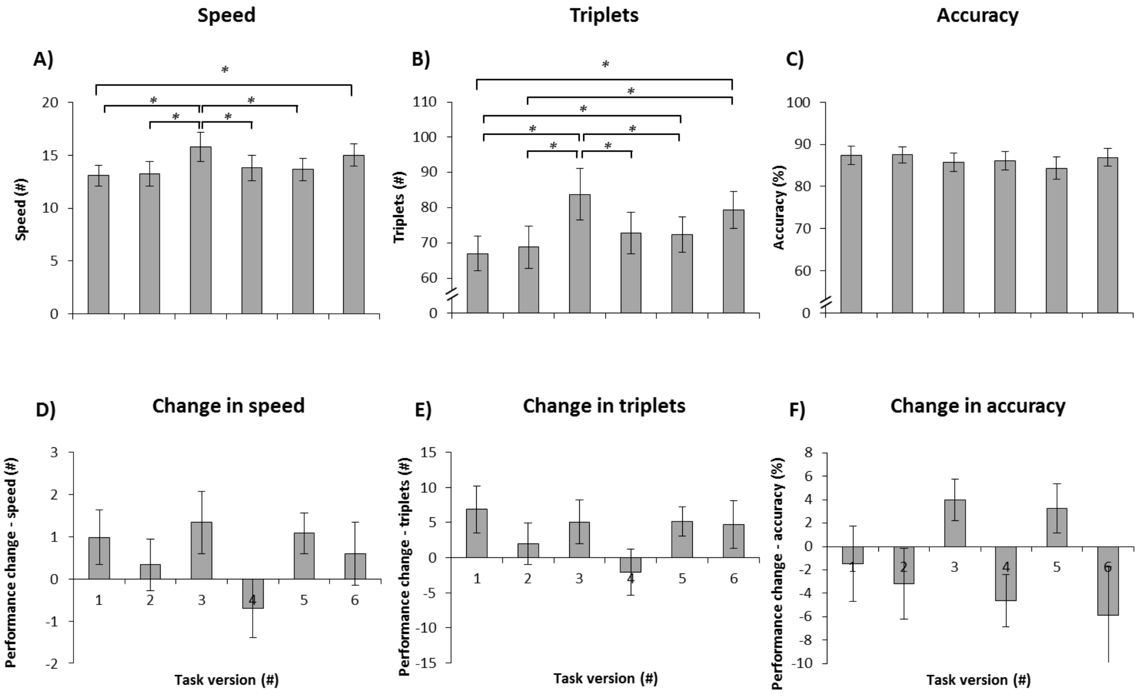

Figure S3. Evaluation of task difficulty for each fingertapping sequence (M ± SE). Note that error bars have been corrected to show within-subject variability. Task difficulty was investigated for the learning condition for each specific sequence for the three main outcome variables: **(A)** speed, **(B)** triplets, and **(C)** accuracy. Results indicated a main learning effect, demonstrated by significant better performance on the last compared with the first version for speed and triplets. In addition, significantly better performance was observed for the third version compared to all other versions. Yet, no significant differences were observed for performance changes for **(D)** speed, **(E)** triplets, or **(F)** accuracy. * $p < 0.050$.

Circadian effects. Considering the seven sessions took place in the early morning and late evening, the possibility of circadian effects on acquisition performance was evaluated. We therefore made within-subject contrasts for performance during learning sessions in the morning and evening for those patients who performed in all six task versions and who therefore had an equal number of learning sessions in the morning and evening ($n = 19$). Importantly, sessions that included a seizure were not included in this investigation. No differences in performance were observed when contrasts were made between learning sessions taking place in the morning or evening for speed ($Z = -0.240$, $p = 0.405$; Figure S5A), triplets ($Z = -0.362$, $p = 0.359$; Figure S5B), or accuracy ($Z = -0.008$, $p = 0.497$; Figure S5C)).

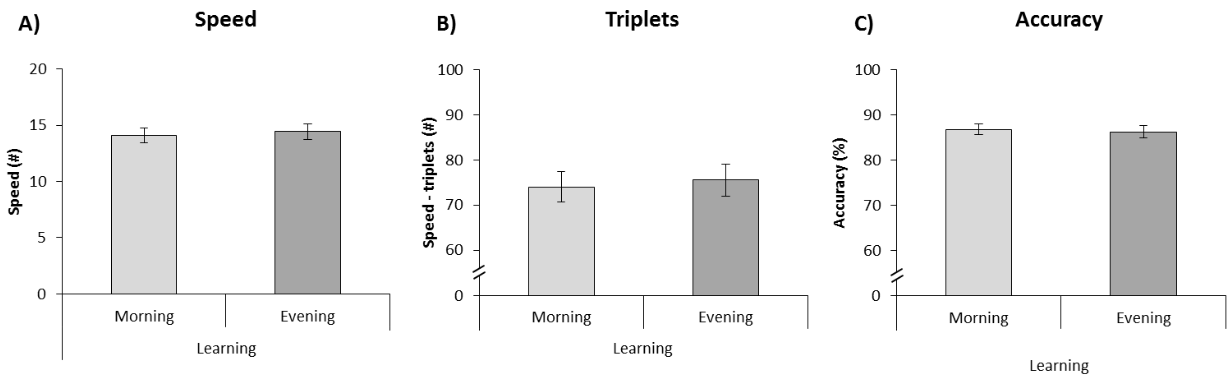

Figure S4. Behavioral performance contrasted between morning and evening learning sessions (M ± SE). Note that error bars have been corrected to show within-subject variability. No differences in performance were observed whether learning was conducted in the morning or evening, as evaluated for **(A)** speed, **(B)** triplets, and **(C)** accuracy.

Medication. Patients' anti-epileptic drug (AED) usage was tailored to individual requirements and therefore varied strongly between patients (for an overview of AEDs used, see Table S1). As the patients were in the EMU for diagnostic purposes, medication was tapered to increase the likelihood of epileptiform activity during their stay. Given the wide variance, the analyses did not take medication or its dosage into account.

Table S1: Patient overview

ID	Gender (M/F)	Age (Y)	DMEQ score	BDI-II score	Age of epilepsy onset	Epilepsy type	Localization	Lateralization	Medication	Lesional MRI	Seizures during assessment
01*	Male	29	47	7	28	Focal secondarily generalized tonic clonic seizures	Fronto-temporal	Left	Trileptal	No	2
02	Female	25	56	5	7	Focal complex; secondarily generalized tonic clonic seizures	Temporal	Left	Lamotregin, Vimpat	No	0
03+	Male	19	51	4	13	Focal simple seizures; secondarily generalized tonic clonic seizures	Temporal	Right	Trileptal	No	27
04	Female	30	44	12	22	Focal simple and focal complex seizures	Temporal	Right	Levebon, Zebenix, Citalopram, Pantoloc	No	0
05*	Male	24	27	12	21	Focal secondarily generalized tonic clonic seizures	Temporo-mesial	Right	Trileptal	No	6
06	Female	34	56	0	17	Generalized tonic-clonic seizures	*Unclear*	*Unclear*	Camictal	No	0
07	Female	45	63	10	NA	generalized tonic clonic with myclonia	Temporal	Right	Aktiferrin, Eutyrox, Hydal, Levetiracetam, Magnonorm, Maxi-Koiz, Pantoloc, Rocatrol, Zonegran	No	0
08	Male	21	52	1	19	Focal complex with secondary generalized tonic clonic seizures	Fronto-temporal	Bilateral	Convulex retard, Triceptal	No	0
09	Female	35	57	5	24	Focal simple & secondary generalized tonic clonic seizures	Unclear	Left	Levebon, Lovenox	No	0
10	Male	56	71	18	17	Secondary	Unclear		Levetiracetam, Lyrika,	No	0

						generalized tonic seizures		Unclear	Plavix, Hydal, Neuromultivit, Frisium		
11*	Male	24	52	14	12	Myoclonic and generalized tonic clonic seizures	Frontal	Left	Durotiv, Cefuroxin, Levetiracetam, Zebivix	No	1
12	Female	26	46	16	18	Primary generalized tonic clonic seizures	Fronto-centro-parietal	*Unclear*	No medication	No	0
…	…	…	…	…	…	…	…	…	…	…	…

Table S1: Patient overview (continued I)

ID	Gender (M/F)	Age (Y)	DMEQ score	BDI-II score	Age of epilepsy onset	Epilepsy type	Localization	Lateralization	Medication	Lesional MRI	Seizures during assessment
13	Female	25	58	2	2	Focal simple and focal secondary generalized tonic clonic seizures	Fronto-temporal	Left	Levetiracetam, Thyrex Levetiracetam	No	0
14	Female	48	71	16	2	Unclear	Temporal	Left	Levetiracetam, Trileptal, Concor, Blopress Plus, Euthyrox, Pantoloc, Sertralin, Seroquel, Halcion, Dominal Forte	No	0
15*	Male	35	60	12	3	Focal secondary generalized tonic clonic seizures	Temporal	Left	Levebon	No	1
16*	Female	19	48	10	19	Focal-complex seizures	Fronto-central	Left	Levetiracetam	No	7
17	Female	62	59	7	55	Focal simple seizures with generalized tonic clonic seizures	Frontal	Bilateral	Levetiracetam	No	0
18	Female	29	54	0	11	Focal simple and complex secondary generalized tonic clonic seizures	Parieto-occipital	Bilateral	Levetiracetam	No	0
19	Male	24	35	12	20	Focal secondary generalized tonic clonic seizures	Frontal	Left	Levetiracetam, Vimpat	Yes	0
20	Female	28	65	12	29	Focal simple and complex,	Temporal	Left	No medication	No	0

						& secondary generalized tonic clonic seizures					
21	Male	18	59	6	6	Generalized tonic clonic seizures	Frontal	Unclear	Levetiracetam	No	0
22	Male	66	54	3	62	Focal simple & complex seizures	Temporal	Right	Tegretol	No	0
23	Female	19	30	19	19	Generalized tonic clonic seizures	Frontal	Left	Levebon	No	0
24*	Female	50	50	5	37	Focal complex with secondary tonic clonic seizures	Fronto-temporal	Unclear	Levetiracetam	No	2
…	…	…	…	…	…	…	…	…	…	…	…

Table S1: Patient overview (continued II)

ID	Gender (M/F)	Age (Y)	DMEQ score	BDI-II score	Age of epilepsy onset	Epilepsy type	Localization	Lateralization	Medication	Lesional MRI	Seizures during assessment
25	Female	48	60	15	43	Generalized tonic clonic seizures	Frontal	Bilateral	Levetiracetam, Amiridex	No	0
26	Male	28	44	0	13	Generalized tonic clonic seizures	Bifrontal	Bilateral	Levetiracetam, Lonegram	No	0
27*	Male	19	44	1	3	Focal simple seizures	posterior- parieto-occipitaler	Right	No medication	No	1
28	Female	54	51	5	18	Focal complex and secondary generalized seizures	Fronto-temporal	Left	Tegretol, Lovebon	No	0
29	Male	20	55	4	20	Idiopathic generalized seizures	Unclear	*Unclear*	Levetiracetam, Zonegran	No	0
30	Male	35	33	0	31	Focal complex and secondary generalized tonic clonic seizures	Temporal	Left	Trileptal, Setralin	No	0
31	Male	25	55	5	13	Idiopathic generalized seizures	Bifrontal	Bilateral	Lamotregin	No	0

32*	Female	23	43	19	5	Focal seizures & secondary generalized tonic clonic seizures	Unclear	Bilateral	Levetiracetam, Vimpat	No	1
33*	Female	19	47	5	19	Focal to bilateral clonic seizures	Temporal	Left	Levetiracetam	No	5
34	Male	22	44	31	18	Focal and focal hypermotoric seizures	Frontal	Bilateral	No antiepileptic medication	No	0
35	Female	56	71	39	35	Partial seizures with focal bilateral tonic clonic seizures	Unclear	*Unclear*	Levetiracetam, lamotrigin	No	0
36	Male	27	42	13	21	Generalized tonic clonic seizures	Unclear	Left	Zonegran, Depakine Chrono	No	0
…	…	…	…	…	…	…	…	…	…	…	…

Table S1: Patient overview (continued III)

ID	Gender (M/F)	Age (Y)	DMEQ score	BDI-II score	Age of epilepsy onset	Epilepsy type	Localization	Lateralization	Medication	Lesional MRI	Seizures during assessment
37	Male	53	39	18	49	Focal bilateral tonic clonic seizures	Unclear	*Unclear*	Levetiracetam	Yes	0
38	Female	30	59	19	9	Focal secondary generalized tonic clonic seizures	Unclear	Unclear	No medication	No	0
39*	Male	26	59	12	15	Focal motor to bilateral tonic clonic seizures	Temporal	Left	Neurotop, Levetiracetam, Fycompa	No	6
40	Female	27	67	0	5	Bilateral tonic clonic seizures	*Unclear*	*Unclear*	Topiramat	No	0
41	Male	45	54	9	43	Focal simple seizures	Temporal	Right	Lamotrigin	No	0

						with secondary induced tonic clonic seizures					
42*	Female	28	45	1	25	Focal complex & secondary generalized tonic clonic seizures	Temporal	Right	Levetiracetam, Vimpat	Yes	1
43	Female	23	50	5	5	Focal seizures	Occipital	Right	Levetiracetam, Tegretol	No	0
44	Female	46	40	35	44	Focal to bilateral tonic clonic seizures	Occipital	Right	Levebon, Thyrex	Yes	0
45*	Female	21	40	1	8	Focal to bilateral tonic clonic seizures	Frontal	Right	Levetiracetam, Carbamazepin	No	2
46*	Female	27	51	10	27	Single focal seizures	Temporal	Right	Levetiracetam	Yes	6
47*	Male	19	47	15	14	Focal to bilateral clonic seizures	Temporo-mesial	Left	Levetiracetam, Multibionta	No	1
48	Female	60	63	1	1	*NA*	Temporo-mesial	Left	Tegretol, Levetiracetam, Rivotril	No	0
…	…	…	…	…	…	…	…	…	…	…	…

Table S1: Patient overview (continued IV)

ID	Gender (M/F)	Age (Y)	DMEQ score	BDI-II score	Age of epileps y onset	Epilepsy type	Localization	Lateralization	Medication	Lesional MRI	Seizures during assessment
49	Female	18	46	7	10	Focal simple seizures	Unclear	*Unclear*	Convulex	No	0
50	Female	26	46	5	21	Idiopathic generalized epilepsy & tonic clonic seizures	Unclear	*Unclear*	Levetiracetam	No	0

51	Male	26	42	8	26	Focal epilepsy	Unclear	Left	Levetiracetam	No	0
52*	Male	61	58	15	59	Secondary bilateral tonic clonic seizures	Temporal	Left	Levetiracetam	No	2
53+	Female	42	50	5	8	Tonic seizures	Unclear	*Unclear*	*Unclear*	No	1

Note: * Patient used for within-subject contrasts; + patient not used for between-subject contrasts; DMEQ = Morningness-Eveningness Questionnaire; BDI-II = Beck Depression Inventory

References:

1. Höller, Y.; Hohn, C.; Schwimmbeck, F.; Plancher, G.; Trinka, E. Effects of Antiepileptic Drug Tapering on Episodic Memory as Measured by Virtual Reality Tests. *Front. Neurol.* **2020**, *11*, 93, doi:10.3389/fneur.2020.00093.
2. Hautzinger, M.; Keller, F.; Kühner, C. Das Beck Depressions Inventar II. Deutsche Bearbeitung und Handbuch zum BDI II. Harcourt Test Services. *Frankfurt* **2006**.
3. Griefahn, B.; Künemund, C.; Bröde, P.; Mehnert, P. Zur Validität der deutschen Übersetzung des Morningness-Eveningness-Questionnaires von Horne und Östberg: The Validity of a German Version of the Morningness-Eveningness-Questionnaire Developed by Horne and Östberg. *Somnologie* **2001**, *5*, 71-80, doi:10.1046/j.1439-054X.2001.01149.x.
4. Walker, M.P.; Brakefield, T.; Morgan, A.; Hobson, J.A.; Stickgold, R. Practice with sleep makes perfect: sleep-dependent motor skill learning. *Neuron* **2002**, *35*, 205-211, doi:10.1016/S0896-6273(02)00746-8.
